# Socioeconomic factors, body mass index and bariatric surgery: a Swedish nationwide cohort study

**DOI:** 10.1186/s12889-019-6585-8

**Published:** 2019-03-04

**Authors:** Ensieh Memarian, Kristina Sundquist, Susanna Calling, Jan Sundquist, Xinjun Li

**Affiliations:** 1 Center for Primary Health Care Research, Lund University, Skåne University Hospital, Region Skåne CRC, Building 28, floor 11, Jan Waldenströms gata 35, 205 02 Malmö, Sweden; 2 Department of Family Medicine and Community Health, Department of Population Health Science and Policy, Icahn School of Medicine at Mount Sinai, New York, USA

**Keywords:** BMI, Bariatric surgery, Socioeconomic status

## Abstract

**Background:**

Bariatric surgery is considered to be the most effective method of weight loss today. The aim of the present Swedish study, which was performed in a country that has universal health care, was to investigate if there is an association between socioeconomic factors and bariatric surgery by taking body mass index (BMI) into account.

**Methods:**

In this prospective cohort study, BMI data were collected for the period 1985–2010 from the Military Service Conscription Register (for men) and from the Medical Birth Register in the first trimester (for women). The follow-up period started in 2005 and continued until 2012. Age-standardized cumulative incidence rates (CR) of bariatric surgery were compared between different BMI groups by considering individual variables. We analyzed the association between the individual variables and bariatric surgery using Cox proportional hazard models.

**Results:**

In the study population of 814,703 women and 787,027 men, a total of 7433 women and 1961 men underwent bariatric surgery. In women, the hazard ratios (HRs) for bariatric surgery were higher for low and middle income and educational levels, compared to high income and educational levels. In men, the highest HR for bariatric surgery was found among those with a high income. The HRs when comparing the different socioeconomic groups in those with BMI > 40 kg/m^2^ showed no significant results, except for middle education in women.

**Conclusion:**

Differences in bariatric surgery between socioeconomic groups were found, favoring those with a low socioeconomic status. However, very few socioeconomic differences were found amongst those who had a BMI > 40 kg/m^2^. This indicates that the Swedish healthcare system seems to have achieved equal access to health care for bariatric surgery.

**Electronic supplementary material:**

The online version of this article (10.1186/s12889-019-6585-8) contains supplementary material, which is available to authorized users.

## Background

Obesity, defined as having a body mass index (BMI) of 30 kg/m^2^ and over, is one of the major modifiable causes of preventable death in the developed world [1, 2]. Conventional interventions for weight loss, such as lifestyle modifications (healthy diet and exercise), have often been ineffective in maintaining a sustained weight loss, particularly in individuals with severe obesity (BMI > 40 kg /m^2^) [3]. Due to the enormous clinical burden and ineffectiveness of conventional interventions against obesity [4, 5], it has been highly needed to develop innovative and cost-effective strategies for the treatment of obesity. Today, bariatric surgery is considered to be the most effective strategy to achieve both sustained weight loss and significant improvements in obesity-related comorbidities and quality of life as well as a reduction in mortality in individuals with obesity. Bariatric surgery has also been proven to be cost-effective [6–8].

The utilization of bariatric surgery has increased worldwide due to both an increasing incidence of sever obesity and the expanding evidence of the beneficial effects of surgery [9]. In Sweden, there has been a large increase in bariatric surgery after 2005 [10], presumably in response to a report from the Swedish Council on Technology Assessment in Health Care [11] and the Swedish Obese Subjects study [12]. In Sweden, individuals are eligible for bariatric surgery if they are refractory to nonsurgical therapy and have severe obesity or a BMI > 35 kg/m^2^ in addition to a major obesity-related comorbidity [13] (the same criteria as those from the National Institutes of Health) [9, 14].

In countries that have private health insurance (e.g. USA), socioeconomic factors may significantly influence which individuals receive bariatric surgery despite medical eligibility [15]. Sweden has a universal healthcare insurance system, which means that financial resources should not be a barrier for obesity surgery. According to Swedish law, the tax-financed health care service should be provided on equal terms for the entire population and those who are in the greatest need of health care should be prioritized [16]. In a previous study of ours, however, we found that individuals with a low socioeconomic status underwent bariatric surgery to a lower extent than those with a higher socioeconomic status [10]. This is despite the higher rates of obesity being among individuals with a low socioeconomic status. However, we did not have access to BMI data in our previous study. In the present study, we have access to individual BMI data from two different nationwide registers for men and women. The aim of the present study was to investigate whether differences in the access to bariatric surgery exist for different socioeconomic groups after accounting for BMI. To the authors’ knowledge, this is the first nationwide study examining potential socioeconomic differences in the access to bariatric surgery after having taken BMI into account.

## Methods

### Data sources

Data used in this study were obtained from nationwide registers [17, 18]. The Swedish Medical Birth Register, which is a register of all pregnancies, prenatal care and birth records for all mothers and children in Sweden since 1973, was used to obtain information about women’s weight, height and BMI. A pregnant woman’s first contact with the health care service is during the first trimester.

The Military Conscription Register, which includes a structured and standardized medical assessment of all Swedish men since 1969, was used to obtain information about men’s weight, height and BMI. Men of foreign citizenship and those with a severe disability were excluded from military service. The Total Population Register, maintained by Statistics Sweden, the Swedish government-owned census bureau, was used to obtain the demographic and socioeconomic variables. Additional linkages in the database included data from the National Cause of Death Register and the Immigration Register (to identify dates of immigration and/or emigration). Information about surgical procedures were obtained from the Swedish Inpatient Register maintained by the National Board of Health and Welfare. Data linkages were performed on the basis of a personal identification number that is assigned to each permanent resident in Sweden for their lifetime. This number was replaced by a serial number for each person to provide anonymity. The initial population for women was 913,288 and for men 922,982, before exclusion.

Exclusion criteria were the following:

- Individuals who were older than 65 years in 2005 (born earlier than 1950) and thus not eligible for bariatric surgery
- Individuals with missing data
- Individuals who emigrated before 2005
- Individuals who died before 2005
- Those with BMI > 90 kg/m^2^ (one person) and BMI < 15 kg/m^2^
- Individuals whose BMI was assessed after the obesity surgery
- Men younger than 18 and older than 27 years at baseline (based on the age distribution of those conscripted)
- Women younger than 18 and older than 39 years at baseline (based on the age distribution of women in the study population)

The final study populations were 814,703 for women and 787,027 for men. Only individuals whose BMI was measured during the period 1985–2010 were included. The follow-up period started on January 1, 2005, and proceeded until first hospitalization for bariatric surgery, death, emigration, or the end of the study period on December 31, 2012.

### Outcome variable

We used the Swedish Inpatient Register to identify those who had undergone bariatric surgery. The Swedish Classification of Operations and Major Procedures was used to identify patients undergoing bariatric surgery: gastroplasty (JDF00-JDF21), gastric bypass (JDF10-JDF-11) and gastric banding (JDF20-JDF21). Since there was a large increase in the number of bariatric surgery procedures after 2005 [10], we used the period between 2005 and 2012 to determine the number of procedures.

### Individual variables

#### Sex

Men and women

#### Age

For men, we included the 98% of men who were aged between 18 and 27 years at the time of conscription of whom 96.5% were 18–20 years old. For women, we included the 98% of women who were aged between 18 and 39 years (i.e., women in childbearing ages). The birth years were 1958–1989 for men and 1950–1989 for women.

#### Family income

Family income was calculated at start of follow up, 2005, as annual family income divided by the number of members in the family. The income calculation was weighted, taking the ages of the family members into account. For example, children were given lower consumption weights than adults. The calculation was performed as follows: the sum of all family members’ incomes was multiplied by the individual’s consumption weight divided by the family members’ total consumption weight [19]. The final variable was calculated as empirical quartiles from the distribution [20] and classified as low, middle-low, middle-high, and high. In our previous study [10], there was an overlap between the middle-low and middle-high income groups. As a result of this, we combined these two groups to create one middle-income group, which consisted of 50% of the population.

#### Educational Attainment

Educational attainment was classified as follows:

I. Low: completion of compulsory school or less (< 9 years)
II. Intermediate: completed or partial high school (10–12 years)
III. High: college and/or university (> 12 years)

***Employment*** was defined as yes or no.

***Marital status*** was classified as married/cohabiting or single (defined as unmarried, divorced or widowed).

#### BMI

Body mass index (BMI) is a simple index of weight-for-height, which is commonly used to classify overweight and obesity in adults. It is defined as a person’s weight in kilograms divided by the square of the person’s height in meters (kg/m^2^) [21].

We divided the population in the following BMI groups according to the WHO classification:

1. BMI < 25 (underweight and normal weight)
2. BMI 25–29.99 (overweight)
3. BMI 30–39.99 (obese class I and II)
4. BMI > 40 (obese class III/severely obese)

Criteria for obesity surgery in Sweden is chronic (> 5 year) obesity class III (BMI > 40 kg/m^2^) or chronic obesity class II and at least one obesity comorbidity, e.g. sleep apnea, diabetes type II.

For men, the time point for the BMI calculation was at conscription for military service. For women, the time point was at the first contact with a maternity clinic during their first pregnancy. There is a time lag between the BMI measurement and obesity surgery. This time lag can lead to changes in BMI. This is probably the explanation why some individuals with normal BMI or BMI 25–29 kg/m^2^ at baseline have undergone surgery later.

Outliers were judged to be incorrect measures and were excluded.

### Statistical analysis

All statistical analyses were performed using STATA version 14.1. Age-standardized cumulative incidence rates (CR) of bariatric surgery were compared between different BMI groups considering the other individual variables. Age was standardized according to the age in the study population. A *p* value of <.05 was considered statistically significant. Estimates were calculated based on a 95% confidence interval (CI).

The associations between the individual variables and bariatric surgery were analyzed with Cox proportional hazards models. Cox proportional hazard models were used to study the association between certain events (in this case bariatric surgery) and the time it takes for this event to occur. In this study, the time period started on January 1, 2005, and proceeded until first hospitalization for bariatric surgery, death, emigration, or the end of the study period on December 31, 2012.

Both a univariate, adjusted for BMI and a multivariate Cox regression model including all variables were calculated.

Interaction tests were performed in order to examine whether the association between BMI and surgery was affected by any of the individual characteristics.

## Results

Tables 1 and 2 show the female (*n* = 814,703) and male (*n* = 787,027) study population included in the study. A total of 7433 women and 1961 men underwent bariatric surgery between 2005 and 2012. Among both women and men, the prevalence of individuals with high income, high education, employment and who were married/cohabiting was highest in the group with BMI < 25 kg/m^2^, whereas the prevalence of individuals with low income, low education, no employment and who were single were highest in the groups with BMI 30–39 kg/m^2^ and > 40 kg/m^2^ .

**Table 1:** Distribution and percentages of the female study population by BMI and the individual characteristics

	Study population, n (% of total population)	Average BMI	BMI < 25 at baseline	BMI 25–29 at baseline	BMI 30–39 at baseline	BMI ≥40 at baseline
Median age = 28, range						
less than median	463,067		342,894	88,294	29,969	1910
More than median	351,636		252,435	73,823	23,636	1742
Study population, n (% of total population)	814,703	23.50	595,329 (73.1%)	162,117 (19.9%)	53,605 (6.6%)	3652 (0.4%)
Operated, n (% of operated)	7433	32.31	843 (11.4%)	2025 (27.2%)	3700 (49.8%)	865 (11.6%)
Family Income, n (% of total)						
• Low (25%)	204,204	23.74	142,812 (69.9%)	44,138 (21.6%)	16,135 (7.9%)	1119 (**0.6%**)
• Middle (50%)	407,104	23.45	299,302 (73.5%)	80,006 (19.7%)	26,013 (6.4%)	1783 (0.4%)
• High (25%)	203,395	23.34	153,306 (**75.3%**)	37,973 (18.7%)	11,457 (5.6%)	750 (0.4%)
Education, n (% of total)						
• Low	63,615 (7.8%)	23.75	43,877 (69.0%)	13,751 (21.6%)	5540 (8.7%)	447 (**0.7%**)
• Middle	163,872 (20.1%)	23.47	118,773 (72.5%)	32,887 (20.1%)	11,421 (6.9%)	791 (0.5%)
• High	587,216 (72.1%)	23.47	432,679 (**73.7%**)	115,479 (19.7%)	36,644 (6.2%)	2414 (0.4%)
Employment, n (% of total)						
• Yes	584,962 (71.8%)	23.43	432,377 (**73.9%**)	114,311 (19.5%)	35,979 (6.2%)	2295 (0.4%)
• No	229,741 (28.2%)	23.65	162,952 (70.9%)	47,806 (20.8%)	17,626 (7.7%)	1357 (**0.6%**)
Marital status, n (% of total)						
• Married/cohabiting	353,317 (43.4%)	23.22	266,645 (**75.5%**)	66,067 (18.7%)	19,472 (5.5%)	1133 (0.3%)
• Single	461,384 (56.6%)	23.70	328,684 (71.2%)	96,050 (20.8%)	34,133 (7.4%)	2519 (**0.6%**)

The highest percentages are in bold and underlined. Follow up for bariatric surgery between 2005 and 2012

All percentages are row percentages except for Study population, second column

**Table 2:** Distribution and percentages of the male study population by BMI and the individual characteristics

	Study population, n (% of total population)	Average BMI	BMI < 25 at baseline	BMI 25–29 at baseline	BMI 30–39 at baseline	BMI ≥40 at baseline
Median age = 22, range						
Less than median	780,930		662,456	94,378	23,160	936
More than median	6097		4465	1337	284	11
Study population, n (% of total population)	787,027	22.27	666,921 (84.7%)	95,715 (12.2%)	23,444 (3.0%)	947 (0.1%)
Operated, n (% of operated)	1961	30.97	258 (13.2%)	661 (33.7%)	920 (46.9%)	122 (6.2%)
Family Income, n (% of total)						
• Low (25%)	196,474	22.48	162,476 (82.7%)	26,537 (13.5%)	7107 (3.6%)	354 (**0.2%**)
• Middle (50%)	394,008	22.28	333,033 (84.5%)	48,473 (12.3%)	12,029 (3.1%)	473 (0.1%)
• High (25%)	196,545	22.05	171,412 (**87.2%**)	20,705 (10.5%)	4308 (2.2%)	120 (0.1%)
Education, n (% of total)						
• Low	59,863 (7.6%)	22.62	47,784 (79.8%)	9013 (15.1%)	2914 (4.9%)	152 (**0.2%**)
• Middle	172,898 (22.0%)	22.29	144,831 (83.8%)	22,321 (12.9%)	5531 (3.2%)	215 (0.1%)
• High	554,266 (70.4%)	22.22	474,306 (**85.6%**)	64,381 (11.6%)	14,999 (2.7%)	580 (0.1%)
Employment, n (% of total)						
• Yes	512,626 (65.1%)	22.18	439,063 (**85.7%**)	59,657 (11.6%)	13,476 (2.6%)	430 (0.1%)
• No	274,401 (34.9%)	22.43	227,858 (83.0%)	36,058 (13.2%)	9968 (3.6%)	517 (**0.2%**)
Marital status, n (% of total)						
• Married/cohabiting	157,229 (20.0%)	22.05	137,731 (**87.6%**)	16,660 (10.6%)	2785 (1.8%)	53 (0.03%)
• Single	629,798 (80.0%)	22.32	529,190 (84.0%)	79,055 (12.6%)	20,659 (3.3%)	894 (**0.1%**)

The highest percentages are in bold and underlined. Follow up for bariatric surgery between 2005 and 2012

All the percentages are row percentages except for Study population, second column

The age-adjusted cumulative rates of bariatric surgery for the different BMI groups and by individual characteristics are presented in Table 3 (women) and Table 4 (men). The highest rates are underlined and in bold. The unadjusted rates of bariatric surgery are presented in Additional files 1 and 2: Tables S1a and S1b.

**Table 3:** Age-adjusted cumulative rates of bariatric surgery (per 1000 individuals), Women

	Study population		BMI 30–39		BMI ≥40	
	Operated (% total population)	Rate, 95% CI	Operated	Rate, 95% CI	Operated	Rate, 95% CI
Study population	7433	9.1 (8.9–9.3)	3700	70.0 (67.8–72.2)	865	237.1 (223.0–251,2)
Family income						
• Low	2224 (1.1%)	9.6 (9.2–10.0)	1186	68.0 (64.0–72.0)	262	225.9 (200.6–251,2)
• Middle	4024 (1.0%)	**9.7 (9.4–10.1)**	1933	**75.0 (71.8–78.3)**	429	242.2 (222.0–262.3)
• High	1185 (0.6%)	7.8 (7.3–8.3)	581	66.1 (59.4–72.7)	174	**281.1 (229.0–333.2)**
Education						
• Low	945 (1.5%)	13.3 (12.3–14.2)	457	78.6 (71.0–86.2)	109	234.0 (192.7–275.2)
• Middle	2420 (1.5%)	**13.8 (13.2–14.3)**	1128	**98.6 (93.0–104.2)**	220	**267.1 (234.9–299.3)**
• High	4068 (0.7%)	7.5 (7.2–7.7)	2115	60.3 (57.8–62.8)	553	227.0 (209.8–244.1)
Employment						
• Yes	4842 (0.8%)	8.6 (8.4–8.9)	2443	**72.0 (69.2–74.8)**	533	238.4 (220.1–256.7)
• No	2591 (1.1%)	**10.4 (10.0–10.8)**	1257	68.0 (64.1–71.8)	332	**238.5 (215.2–261.9)**
Marital Status						
• Married/cohabiting	3112 (0.9%)	9.1 (8.7–9.4)	1507	**81.9 (77.9–85.9)**	274	**248.3 (221.5–275.1)**
• Single	4321 (0.9%)	9.1 (8.9–9.4)	2193	63.7 (61.1–66.3)	591	233.2 (216.5–249.8)

The different BMI groups and the individual characteristics. Followed for bariatric surgery between 2005 and 2012 (the highest rates are in bold and underlined)

**Table 4:** Age-adjusted cumulative rates of bariatric surgery (per 1000 individuals), Men

	Study population		BMI 30–39		BMI ≥40	
	Operated (% total population)	Rate, 95% CI	Operated	Rate, 95% CI	Operated	Rate, 95% CI
Study population	1961	2.5 (2.4–2.6)	920	39.2, (36.8–41.8)	122	128.1 (106.8–149.3)
Family income						
• Low	490 (0.25%)	2.5 (2.3–2.7)	245	34.5 (30.3–38.8)	38	107.9 (75.5–140.3)
• Middle	1090 (0.28%)	**2.8 (2.6–2.9**)	499	**41.5 (38.0–45.1)**	70	**145.9 (114.2–177.6)**
• High	381 (0.19%)	1.9 (1.7–2.1)	176	41.0 (35.0–47.0)	14	112 (55.6–168.9)
Education						
• Low	316 (0.53%)	**5.3 (4.7–5.9)**	154	52.6 (44.4–60.7)	23	**151.1 (94.3–207.9)**
• Middle	811 (0.47%)	4.7 (4.4–5.0)	351	**64.1 (57.6–70.6)**	26	121.1 (77.4–151.0)
• High	834 (0.15%)	1.5 (1.4–1.6)	415	27.7 (25.1–30.3)	73	124.3 (97.5–151.0)
Employment						
• Yes	1226 (0.24%)	2.4 (2.3–2.5)	574	**42.7 (39.3–46.1)**	57	**129.7 (98.0–161.4)**
• No	735 (0.27%)	**2.7 (2.5–2.9)**	346	34.8 (31.2–38.4)	65	127.1 (98.4–155.8)
Marital Status						
• Married	465 (0.30%)	**3.0 (2.7–3.2)**	186	**68.8 (59.2–78.4)**	9	**150.4 (50.0–250.7)**
• Single	1496 (0.24%)	2.4 (2.3–2.5)	734	35.5 (33.0–38.1)	113	126.2 (104.5–148.0)

Different BMI groups and individual characteristics. Followed for bariatric surgery between 2005 and 2012 (the highest rates are in bold and underlined)

Table 5 shows hazard ratios (HRs) for bariatric surgery in the women and men, respectively, by the individual characteristics in three different models, where model 1 is univariate, model 2 is adjusted for BMI and model 3 is multivariate (adjusted for all the included variables, i.e. BMI, income, education, employment and marital status). In women, the HRs for bariatric surgery were higher for low and middle levels of income and education compared to high income and educational levels. The HRs were also higher for those who were employed and/or were married/cohabiting. In men, low and middle family income was associated with higher HRs only in model 1. After controlling for the other individual factors, the highest HR for bariatric surgery was found among those with a high income. Low and middle levels of education were strongly associated with bariatric surgery; e.g. the HR was 2.6 [95% CI: 2.4–2.9] for a middle educational level. As in women, the highest HRs were found in those who were employed and/or were married/cohabiting.

**Table 5:** Unadjusted and adjusted hazard ratios for bariatric surgery by the individual characteristics

	Univariate, model 1		Adjusted for BMI, model 2		Multivariate, model 3	
	Hazard ratio, 95% CI	*P* value	Hazard ratio, 95% CI	*P* Value	Hazard ratio, 95% CI	*P* value
Women						
Family income						
• Low	**1.9 (1.8–2.0)**	0.001	**1.6 (1.5–1.7)**	0.001	**1.4 (1.3–1.5)**	0.001
• Middle	1.7 (1.6–1.8)	0.001	1.5 (1.4–1.6)	0.001	**1.4 (1.3–1.5)**	0.001
• High	1.00		1.00		1.00	
Education						
• Low	**2.2 (2.0–2.3)**	0.001	1.8 (1.6–1.9)	0.001	1.7 (1.5–1.8)	0.001
• Middle	2.1 (2.0–2.2)	0.001	**2.1 (2.0–2.2)**	0.001	**2.1 (2.0–2.1)**	0.001
• High	1.00		1.00		1.00	
Employment						
• Yes	1.00		1.00		1.00	
• No	**1.4 (1.3–1.5)**	0.001	**1.1 (1.0–1.2)**	0.001	0.9 (0.9–0.9)	0.010
Marital Status						
• Married	1.00		1.00		1.00	
• Single	**1.1 (1.0–1.1)**	0.001	0.9 (0.9–0.9)	0.001	0.9 (0.9–0.90)	0.010
Men						
Family income						
• Low	1.3 (1.1–1.5)	0.001	0.9 (0.8–1.0)	0.050	0.8 (0.7–0.9)	0.020
• Middle	**1.4 (1.3–1.6)**	0.001	1.1 (1.0–1.2)	0.110	1.0 (0.9–1.2)	0.740
• High	1.00		1.00		1.00	
Education						
• Low	**3.5 (3.1–4.0)**	0.001	2.2 (1.9–2.5)	0.001	2.1 (1.9–2.4)	0.001
• Middle	3.1 (2.8–3.4)	0.001	**2.8 (2.5–3.1)**	0.001	**2.6 (2.4–2.9)**	0.001
• High	1.00		1.00		1.00	
Employment						
• Yes	1.00		1.00		1.00	
• No	**1.1 (1.0–1.2)**	0.020	0.8 (0.8–0.9)	0.001	1.0 (0.9–1.1)	0.320
Marital Status						
• Married	1.00		1.00		1.00	
• Single	0.8 (0.7–0.9)	0.001	0.5 (0.5–0.6)	0.001	0.6 (0.5–0.7)	0.001

Cox regression analysis with univariate, adjusted for BMI, and multivariate models (the highest hazard ratios are in bold and underlined)

We estimated the HRs for the different individual characteristics stratified by BMI to further analyze the association between socioeconomic factors and bariatric surgery. The results are presented in Table 6, which shows the results, stratified for those with BMI > 40 kg/m^2^ and BMI 30–39 kg/m^2^, in multivariate models. In those with BMI > 40 kg/m^2^, none of the socioeconomic variables were associated with bariatric surgery, except for middle educational level in women. In those with BMI 30–39 kg/m^2^, women had higher HRs for low and middle family income and education. Those women who were single had a lower HR than those who were married/cohabiting. For men, the HR was significantly higher for low and middle education. The same analysis was conducted for individuals with BMI 35–39 kg/m^2^; however, the results were almost unchanged compared to the results in Table 6 for BMI > 40 kg/m^2^ (data not presented in tables). As an additional analysis, we included age as a confounder in Table 6 and the results remained almost identical.

**Table 6:** Hazard ratios for bariatric surgery by the individual characteristics, stratified for BMI

	Women BMI 30–39 kg/m^2^		Men BMI 30–39 kg/m^2^		Women BMI > 40 kg/m^2^		Men BMI > 40 kg/m^2^	
	Hazard ratio 95% CI	*P* Value	Hazard ratio 95% CI	*P* Value	Hazard ratio 95% CI	*P* Value	Hazard ratio 95% CI	*P* Value
Family income								
Low	**1.3 (1.2–1.5)**	**< 0.001**	0.8 (0.7–1.0)	0.085	1.0 (0.8–1.2)	0.889	0.9 (0.4–1.7)	0.660
Middle	**1.4 (1.3–1.5)**	**< 0.001**	1.0 (0.8–1.2)	0.845	1.0 (0.8–1.2)	0.839	1.3 (0.7–2.23	0.411
High	1.00		1.00		1.00		1.00	
Education								
Low	**1.4 (1.3–1.6)**	**< 0.001**	**1.9 (1.6–2.3)**	**< 0.001**	1.1 (0.9–1.3)	0.611	1.2 (0.7–1.9)	0.376
Middle	**1.7 (1.6–1.8)**	**< 0.001**	**2.2 (1.9–2.5)**	**< 0.001**	**1.3 (1.1–1.5)**	**0.005**	0.9 (0.6–1.4)	0.193
High	1.00		1.00		1.00		1.00	
Employment								
Yes	1.00		1.00		1.00		1.00	
No	1.0 (0.9–1.1)	0.710	1.0 (0.8–1.1)	0.604	1.1 (0.9–1.2)	0.370	1.1 (0.7–1.6)	0.184
Marital Status								
Married/cohabiting	1.00		1.00		1.00		1.00	
Single	**0.8 (0.8–0.9)**	**< 0.001**	**0.6 (0.5–0.7)**	**< 0.001**	0.9 (0.8–1.1)	0.322	0.7 (0.3–1.4)	0.315

Cox regression analysis, multivariate models, women and men with BMI 30–39 kg/m^2^ and BMI > 40 kg/m^2^ (significant p value are in bold and underlined)

The interaction test between bariatric surgery and BMI was performed considering the different individual characteristics. The interactions that were statistically significant are presented in the Additional files 3: Figure S1 and Additional files 4: Figure S2. Except for marital status in men, we did not find a clear pattern in the potentially differential effects of SES on bariatric surgery. Thus, the clinical significance of these interactions is unclear. The effect of BMI on surgery for men was modified by marital status, i.e. those who were married had a higher rate of surgery.

## Discussion

The main findings of the present study are that socioeconomic differences exist between individuals who receive bariatric surgery in Sweden and those who do not. However, in severely obese individuals (BMI > 40 kg/m^2^), these differences disappeared with the exception of women with middle educational level who had higher rates of surgery. Sweden has universal health care, which enable individuals with BMI > 40 kg/m^2^ to receive publicly funded bariatric surgery. This might be the explanation to diminishing socioeconomic differences between individuals with BMI > 40 kg/m^2^. A study by Krajewski et al. showed that access to emergency operative care was related to SES in the United States where most individuals have private health insurance, whereas in Canada, with universal health insurance, no such relationship was found [22].

In 2006, almost 1500 individuals underwent bariatric surgery in Sweden. According to previous estimations, there were 10,000–15,000 individuals who could be eligible for surgery [23]. This indicates that bariatric surgery access may not meet the demand due to factors beyond SES, such as prioritization in healthcare utilization.

We hypothesized that the potential effect of socioeconomic factors was higher for individuals with BMI < 40 kg/m^2^, who are not eligible for publicly funded bariatric surgery unless BMI is ≥35 kg/m^2^ in addition to the presence of an obesity-related comorbidity. These individuals might receive bariatric surgery in case they are able to finance the surgery with their own financial resources. Nevertheless, the results of this study show that women with low and middle family income and education and men with low and middle education had higher HRs for bariatric surgery. This might be explained by the fact that we did not have access to all data concerning privately paid bariatric surgeries. It is possible that some inequality in SES exists for those with BMI < 40 kg/m^2^.

In the present study, the HRs for bariatric surgery were higher for low and middle income and educational levels for women even when adjusted for BMI and the other variables. Similarly, the HRs for men were higher for low and middle educational groups even when adjusted for all other variables. These results complement findings from our previous study [10] where the HRs were higher for those with low and middle income and educational level. One limitation of our earlier study [10] was that we did not have access to BMI data, which was overcome in the current study. According to another Swedish study, the increase in obesity between 2000 and 2012 was steepest among those with a middle educational level and, in 2012, the prevalence of obesity was almost twice as high among those with a low or middle educational level compared with those with a high educational level [24].

An Australian study showed that bariatric surgery rates were higher among those with higher SES [25]. In contrast, our earlier study indicated that individuals with high income and education underwent bariatric surgery to a lower extent, which we interpreted was due to the lower prevalence of obesity in this group. According to the current study, however, the HRs were also lower among those with a high income and educational level (for women) and those with a high educational level (for men), whereas the HR in men was lowest among those with a low income. This socioeconomic difference disappeared, however, when we examined those with a BMI > 40 kg/m^2^. The reasons behind our results are not possible to disentangle in the present study but some explanations behind the lower HRs for bariatric surgery among individuals with high SES are plausible. The first explanation is that those with high income may be able to pay for their surgery with private resources even if they are not eligible for publicly funded surgery according to national guidelines. Not all individuals that pay for the surgery with private resources might be included in our data. Another explanation is that those with a high income and/or education may have fewer comorbidities [26], use alternative methods for weight reduction and/or are more aware of complications of surgery for why they opt out this alternative. Another explanation might be the time lag between the BMI measurement and the surgery, which could mean that those with a high education and/or income succeeded to lose weight and were therefore not eligible or in need of surgery. However, a Swedish longitudinal study showed that BMI increased in all age groups in Sweden between 1980 and 2005, even after controlling for education [27]. That study also showed that the largest increase in BMI was in young and middle-aged individuals, which are the individuals included in the present study.

It seems that the patterns of socioeconomic characteristics differ between men and women except for education, which shows a higher HR for surgery in the middle educational group for both men and women; this confirms the results of our previous study [10]. Differences in socioeconomic characteristics between men and women might be explained by several factors. Women usually get operated on at an earlier age [10] and might have other reasons for accepting/demanding the surgery such as cosmetic and fertility reasons whereas men usually undergo the operation later in life and perhaps mostly due to comorbidities. Both married/cohabiting men and women had a slightly higher HR for surgery. This may be due to the encouraging role of their spouses to get treatment for obesity. Several studies have shown a positive effect of marriage/cohabiting on health and longevity and these benefits of marriage may sometimes be higher for men than for women [28].

Previous studies have shown significant disparities in socioeconomic characteristics between the severely obese population and the subgroup that actually receive bariatric surgery [9, 15, 29]. In countries with a private healthcare system, some of these variations might be explained by financial inequalities. However, in countries with a publicly funded system, no obvious reason for these disparities are apparent [30, 31]. Few studies have systematically analyzed the factors that cause the variation in receiving bariatric surgery. It is generally assumed that much of the variation is explained by socioeconomic barriers. An American study did not support this assumption; it instead emphasized the patients’ perspective [32]. In that study, men were less likely than women and African Americans were less likely than Caucasians to have seriously considered bariatric surgery after accounting for sociodemographic factors. Having a lower ideal weight was also associated with having considered bariatric surgery, which partly explains the gender differences. Men may be less likely than women to consider bariatric surgery, which may be due to them being less concerned about the negative impact of extreme obesity on health [30]. A Chinese study showed that gluttonous behaviors were positively correlated with the acceptance level of bariatric surgery [33]. Physicians’ recommendations were also a strong independent factor for patients considering bariatric surgery according to an American study [32]. This study also found that men and African Americans were less likely to be recommended bariatric surgery by their doctors. The results of the present study indicate that the Swedish healthcare system has achieved its goal of equal health care for the entire population regarding bariatric surgery, since those with BMI > 40 kg/m^2^ were not affected by socioeconomic factors in their likelihood to receive surgery, and there was no strong association between socioeconomic characteristics and the rate of bariatric surgery. In addition, the socioeconomic differences favored those with a low and middle socioeconomic status rather than those with a high socioeconomic status.

### Limitations and strengths

This study has some limitations. The most important limitation is the time lag between the BMI measurement and the obesity surgery, which means that we had no access to the BMI data at surgery (Additional file 2, Additional file 3). Information about SES was collected after 2005 and close in time to the bariatric surgery. However, SES could change over time and the impact of potential changes in SES on BMI during these years is therefore unclear. However, as mentioned above, previous research has found that BMI increased in all age groups in Sweden during the study period [27] and BMI early in adulthood is a good predictor of BMI later in life. It is also unclear how many in the BMI group 1–3 that actually became severely obese during the study period and thus eligible for surgery. For women, we only included the population that had a pregnancy, thus not including those who did not become pregnant. An additional limitation is that the women and men were not directly comparable as most men completed their military service between the ages of 18–20 years (96.5%) whereas the women’s childbearing age was mainly between 18 and 39 years. Furthermore, pregnant women will likely have a higher BMI. Finally, socioeconomic variables cannot fully measure socioeconomic status.

However, the limitations of this study are balanced by its strengths. To our knowledge, the current study is the first nationwide study that considers socioeconomic characteristics in relation to rates of obesity surgery, including levels of BMI. An additional strength is that we had access to a large database, which included all publicly funded surgeries, and the main purpose was to examine whether the publicly funded surgery is equally distributed, irrespective of socioeconomic status. Finally, both men and women were included in the study and analyzed separately. According to the Swedish National Board of Health and Welfare, only a small percentage of records (0.5–3.0%) of all infants are missing from the Swedish Medical Birth Register [34].

## Conclusion

The present nationwide study shows differences between socioeconomic groups and rates of bariatric surgery, favoring those with a low socioeconomic status. However, socioeconomic differences disappeared in those individuals with a BMI > 40 kg/m^2^, which indicates that severe obesity rules out socioeconomic differences in bariatric surgery rates. The Swedish healthcare system seems to have achieved its goal of equal health care for the entire population regarding bariatric surgery.

## Additional files

Additional file 1: **Table S1.** a. Rate of bariatric surgery (per 1000 individuals) for different BMI group, by individual characteristics, closed cohort, women. (DOC 35 kb)

Additional file 2: **Table S1.** b. Rate of bariatric surgery (per 1000 individuals) for different BMI group, by individual characteristics, closed cohort, men. (DOC 36 kb)

Additional file 3: **Figure S1.** Interaction between BMI and other variables, women. (JPG 60 kb)

Additional file 4: **Figure S2.** Interaction between BMI and other variables, men. (JPG 76 kb)

## Abbreviations

BMI: Body mass index
CI: Confidence interval
CR: Age standardized cumulative incidence rate
HR: Hazard ratio
SCB: Statistics Sweden
SES: Socioeconomic status
WHO: World Health Organization
