# Supplementary material for: Socioeconomic factors, body mass index and bariatric surgery: a Swedish nationwide cohort study
Source: BMC Public Health. 2019 Mar 4;19:258. doi: 10.1186/s12889-019-6585-8 (PMC6399907; doi:10.1186/s12889-019-6585-8)
Supplement: Supplementary file 1 — Table S1. a. Rate of bariatric surgery (per 1000 individuals) for different BMI group, by individual characteristics, closed cohort, women. (DOC 35 kb) [file 12889_2019_6585_MOESM1_ESM.doc]

**Table S1a, Rate of bariatric surgery (per 1000 individuals) for different BMI group, by individual characteristics, closed cohort, Women.**

|  | **Total population** | | **BMI 30-39** | | **BMI ≥40** | |
| --- | --- | --- | --- | --- | --- | --- |
|  | **Operated**  **(% of total)** | **Rate, (CI)** | **Operated** | **Rate, (CI)** | **Operated** | **Rate, (CI)** |
| **Total population** (815,554) | 7433 | 9.1 (8.9-9.3) | 3700 | 69.0, (66.8-71.3) | 865 | 236.9 (221.6-253.2) |
| **Family income**   - **Low** - **Middle** - **High** | 2224 (1.1%)  4024 (1.0%)  1185 (0.6%) | **10.9 (10.4-11.3)**  9.9 (9.6-10.2)  5.8 (5.5-6.2) | 1186  1933  581 | 73.5 (69.4-77.8)  **74.3 (71.1-77.7)**  50.7 (46.8-55.0) | 262  429  174 | 234.1 (207.4-264.3)  **240.6 (218.9-264.5)**  232.0 (200.0-269.2) |
| **Education**   - **Low** - **Middle** - **High** | 945 (1.5%)  2420 (1.5%)  4068 (0.7%) | **14.9 (13.9-15.8)**  14.8 (14.2-15.4)  6.9 (6.7-7.1) | 457  1128  2115 | 82. (75.3-90.4)  **98.8 (93.2-104.7)**  57.7 (55.3-60.2) | 106  218  541 | 237.1 (196.0-286.9)  **275.6 (241.3-314.7)**  224.1 (206.0-243.8) |
| **Employment**   - **Yes** - **No** | 4842 (0.8%)  2591 (1.1%) | 8.3 (8.0-8.5)  **11.3 (10.9-11.7)** | 2443  1257 | 67.9 (65.3-70.6)  **71.3 (67.5-75.4)** | 533  332 | 232.2 (213.3-252.8)  **244.7 (219.7-272.4)** |
| **Marital Status**   - **Married** - **Single** | 3112 (0.9%)  4321 (0.9%) | 8.8 (8.5-9.1)  **9.4 (9.1-9.6)** | 1507  2193 | **77.4 (73.6-81.4)**  64.2 (61.6-67.0) | 274  591 | **241.8 (214.8-272.2)**  234.6 (216.4-254.3) |
