# Supplementary material for: Socioeconomic factors, body mass index and bariatric surgery: a Swedish nationwide cohort study
Source: BMC Public Health. 2019 Mar 4;19:258. doi: 10.1186/s12889-019-6585-8 (PMC6399907; doi:10.1186/s12889-019-6585-8)
Supplement: Supplementary file 2 — Table S1. b. Rate of bariatric surgery (per 1000 individuals) for different BMI group, by individual characteristics, closed cohort, men. (DOC 36 kb) [file 12889_2019_6585_MOESM2_ESM.doc]

**Table S1b, Rate of bariatric surgery (per 1000 individuals) for different BMI group, by individual characteristics, closed cohort, Men.**

|  | **Total population** | | **BMI 30-39** | | **BMI ≥40** | |
| --- | --- | --- | --- | --- | --- | --- |
|  | **Operated**  **(% total population)** | **Rate,(CI)** | **Operated** | **Rate, (CI)** | **Operated** | **Rate, (CI)** |
| **Total population** (781,071) | 1961 | 2.5 (2.4-2.6) | 920 | 39.2 (36.8-41.9) | 122 | 130.0 (110.0-150.0) |
| **Family income**   - **Low** - **Middle** - **High** | 490 (0.3%)  1,090 (0.3%)  381 (0.2%) | 2.5 (2.3-2.7)  **2.8 (2.6-2.9)**  1.9 (1.8-2.1) | 245  499  176 | 34.5 (30.4-39.1)  **41.5 (38.0-45.2)**  40.9 (35.2-47.4) | 38  70  14 | 107.3 (78.1-147.5)  **148.0 (117.1-187.1)**  116.7 (69.1-197.0) |
| **Education**   - **Low** - **Middle** - **High** | 316 (0.5%)  811 (0.5%)  834 (0.2%) | **5.3 (4.7-5.9)**  4.7 (4.4-5.0)  1.5 (1.4-1.6) | 154  351  415 | 52.9 (45.1-61.9)  **63.5 (57.2-70.5)**  27.7 (25.1-30.5) | 23  26  73 | **151.3 (100.6-227.7)**  120.9 (82.3-177.6)  125.9 (100.1-158.3) |
| **Employment**   - **Yes** - **No** | 1226 (0.2%)  735 (0.3%) | 2.4 (2.3-2.5)  **2.7 (2.5-2.9)** | 574  346 | **42.6 (39.3-46.2)**  34.7 (31.2-38.6) | 57  65 | **132.6 (102.3-171.9)**  125.7 (98.6-160.3) |
| **Marital Status**   - **Married** - **Single** | 465 (0.3%)  1496 (0.2%) | **3.0 (2.7-3.2)**  2.4 (2.3-2.5) | 186  734 | **66.8 (57.9-77.1)**  35.5 (33.1-38.2) | 9  113 | **169.8 (88.4-326.4 )**  126.4 (105.1-152.0) |
