# Supplementary figures and images for: Socioeconomic factors, body mass index and bariatric surgery: a Swedish nationwide cohort study
Source: BMC Public Health. 2019 Mar 4;19:258. doi: 10.1186/s12889-019-6585-8 (PMC6399907; doi:10.1186/s12889-019-6585-8)

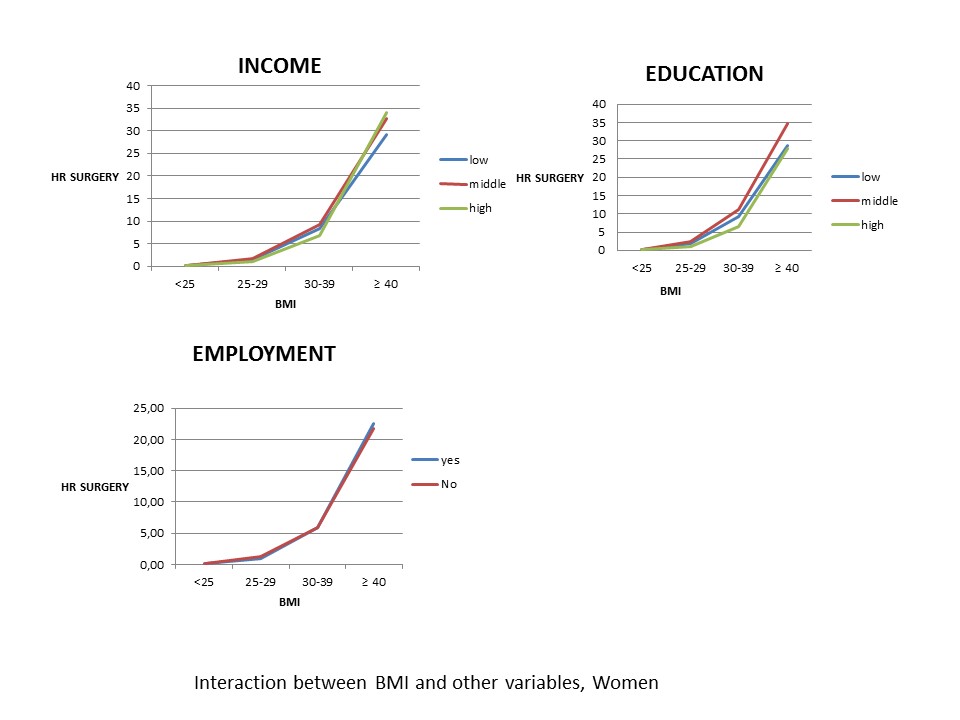

Supplement: Supplementary file 3 — Figure S1. Interaction between BMI and other variables, women. (JPG 60 kb) [file 12889_2019_6585_MOESM3_ESM.jpg]

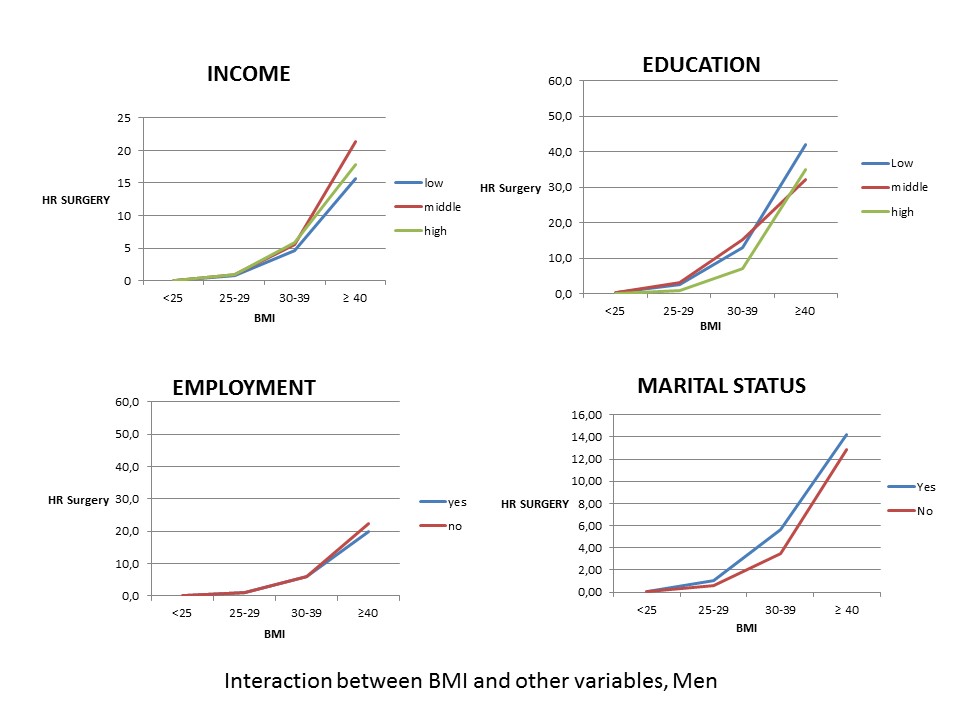

Supplement: Supplementary file 4 — Figure S2. Interaction between BMI and other variables, men. (JPG 76 kb) [file 12889_2019_6585_MOESM4_ESM.jpg]
